# Early Diagnosis of Gallbladder Carcinoma: An Algorithm Approach

**DOI:** 10.5402/2013/239424

**Published:** 2012-10-18

**Authors:** Abhishek Vijayakumar, Avinash Vijayakumar, Vijayraj Patil, M. N. Mallikarjuna, B. S. Shivaswamy

**Affiliations:** ^1^Department of General Surgery, Victoria Hospital, Bangalore Medical College and Research Institute, Bangalore 560002, India; ^2^Department of Radiology, Banaras Hindu University, Uttar Pradesh, Varanasi 221005, India

## Abstract

Gall bladder carcinoma is the most common biliary tract cancer. Delayed presentation and early spread of tumor make it one of the lethal tumors with poor prognosis. Considering that simple cholecystectomy for T1 disease could offer a potential cure, it is increasingly needed to identify it at early stages. Identification of high-risk cases and offering prophylactic cholecystectomy can decrease the incidence of gallbladder carcinoma. With advances in diagnostic tools like contrast-enhanced endoscopic ultrasound, elastography, multidetctor CT, MRI, and PET scan, we can potentially diagnose gallbladder carcinoma at early stages. This paper reviews the various diagnostic modalities available and an algorithmic approach to early diagnosis of gallbladder carcinoma.

## 1. Introduction

Gallbladder carcinoma (GBC) is the most common biliary tract cancer, accounting for 3% of all tumors [1]. GBC is hard to detect and diagnose in its early stages because it usually has very slight symptoms or is asymptomatic. But once the diagnosis is confirmed, most of these patients often have metastasis and invasion. Furthermore, GBC is not sensitive to radiotherapy and chemotherapy. All of these characteristics make GBC a highly lethal tumor with a 5-year survival rate of less than 5% [2]. Considering that survival after simple cholecystectomy for T1 disease is reported to be near 100% [3]. It becomes increasingly necessary for early diagnosis and identifing patients at high-risk of carcinoma and offer them prophylactic cholecystectomy.

The prevalence of gallbladder cancer (GBC) shows great geographical variation. It is rare in the Western world, including the USA, UK, Canada, Australia, and New Zealand, where the incidence rates range between 0.4 and 0.8 in men and between 0.6 and 1.4 in women per 100 000 population. However, high incidence rates, up to 2–4 in men and up to 4–6 in women, have been reported from various countries in central and south America, central and eastern Europe, and Japan. Though the overall age-adjusted incidence rates of GBC in India are low (1.0 for men and 2.3 for women per 100 000 population), the incidence in women in Delhi in north India and Bhopal in central India is as high as 6.6 and 5.2, respectively, much higher than 0.6 in Chennai, and 0.8 in Bangalore in south India. In Delhi, GBC (incidence rate 6.6) was the fourth most common cancer (following cervix, breast, and ovary; incidence rates being 30.1, 28.3, and 8.7, resp.) and the most common gastrointestinal cancer in women (commoner than oesophagus 4.6, stomach 2.4, and colon 2.0) [4].

Risk factors for this neoplasm include gallstones and a history of chronic cholecystitis and an estimated 22% of patients with porcelain gallbladder will develop carcinoma. Others risk factors include choledochal cysts, anomalous pancreaticobiliary duct junctions, and gallbladder polyps > 1 cm in size. Gallbladder carcinoma has a peak incidence in the sixth and seventh decades of life and is three to five times more predominant in females.

Imaging modalities used in evaluating gallbladder diseases include ultrasonography, endoscopic ultrasonography, computer tomography, and MRI.

Gallbladder carcinoma may appear at any of these imaging techniques as a mass completely occupying or replacing the gallbladder lumen, focal or diffuse asymmetric gallbladder wall thickening, or an intraluminal polypoid lesion. 

## 2. Mass Occupying or Replacing the Gallbladder Lumen

Occuring in about 40–65%, on sonography, CT, or MRI, the presence of a large gallbladder mass that nearly fills or replaces the lumen, often directly invading the surrounding liver parenchyma, is highly suggestive of gallbladder carcinoma. Sonography, heterogeneous, and predominantly hypoechoic tumor fills much or all of the gallbladder lumen. Anechoic foci of trapped bile or necrotic tumor may be present, as well as echogenic shadowing foci from gallstones, porcelain gallbladder, or tumor calcifications [5]. Primary gallbladder carcinoma is usually hypodense on unenhanced CT, with up to 40% of lesions showing hypervascular foci of enhancement equal to or greater than that of liver after i. v. contrast administration [6]. On MRI, gallbladder carcinoma usually shows hypo- to isointense signal characteristics on T1-weighted and moderately hyperintense signal characteristics on T2-weighted sequences [7]. 

## 3. Focal or Diffuse Asymmetric Wall Thickening

Gallbladder carcinoma may present as focal or diffuse asymmetric wall thickening in 20–30% of cases. Differentiating between commonly observed causes of diffuse gallbladder wall thickening (Figure 2) such as chronic cholecystitis, acute cholecystitis, gallbladder carcinoma, and other nonspecific causes such as ascites, congestive heart failure, and hypoalbuminemia can be difficult. 

Mitake et al. [8] reported the effectiveness of endoscopic ultrasonography in the diagnosis of gallbladder carcinoma and determination of the extent of tumor invasion; differential diagnosis between early and advanced-stage tumors was 79.5% accurate, and the overall accuracy for tumor invasion depth was 76.9%. Tumor infiltration can be detected as hypoechoic tumor invading the layers of the gallbladder wall. In 1998, Hirooka et al. [9] reported that in contrast-enhanced endosonography, enhancement was observed in 11 of 12 adenocarcinoma (91.7%) but not in adenosquamous carcinoma or cholesterol polyps. Depth of tumor invasion was assessed accurately in 11 of 14 cases (78.6%) by conventional endoscopic ultrasonography, and in 13 of 14 cases (92.9%) by contrast-enhanced endosonography.

Studies have been done using multidetector row CT (MDCT) with a dual-phase technique to show differential gallbladder wall enhancement for distinguishing between benign and malignant causes of gallbladder wall thickening, with reported sensitivity and specificity of 82.5% and 75.9%, respectively. In a study by Kim et al. [10], MDCT findings of hyper enhancing thick inner wall ≥ 2.6 mm during the portal venous phase, weakly enhancing or nonenhancing thin outer wall ≤ 3.4 mm, and irregular and focal wall thickening indicate a malignant cause of flat gallbladder wall thickening rather than benign disease. 

Real-time elastography using acoustic radiation force impulse (ARFI) is a new emerging technique, which uses high intensity focused ultrasound to evaluate the tissue stiffness in the liver, breast, and other organs [11]. It has also been shown to differentiate between benign and malignant nodules in various organs [12]. The likely reason for this difference is that malignant tissues have much higher stiffness due to increased cell density compared to tissues with chronic inflammation and fibrosis.

In a study by Kapoor et al. [13], a mean shear wave velocity of 3.41 m/s (95% confidence interval, 3.1–3.7 m/s) was seen in patients with gallbladder carcinoma. At a cut-off value of 2.7 m/s, elastography showed overall accuracy of 92.8% with sensitivity and specificity of 100% and 91.3%, respectively, for diagnosing gallbladder carcinoma. It had a high likelihood ratio of 11.7, and a false positive rate of 8.5% which was mainly formed by cases of acute cholecystitis.

Thus, routine use elastography during ultrasonography to evaluate increased gallbladder wall thickness combined with MDCT and contrast-enhanced endoscopic ultrasound can help in early diagnosis and staging of diffuse wall thickening type of gallbladder carcinoma.

## 4. Gallbladder Polyps

**Figure 1:**
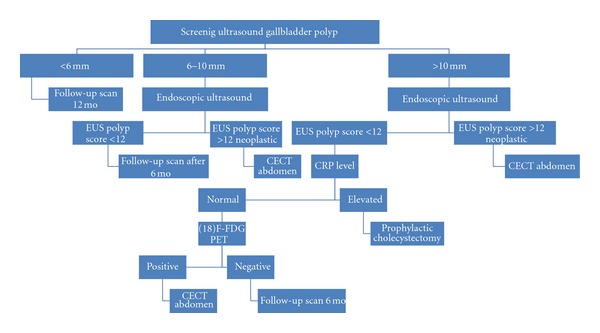
Algorithm for management of gall bladder polyp. EUS (endoscopic ultrasound) CECT (contrast-enhanced computer tomography) FDG PET (fludeoxyglucose positron emission tomography).

**Figure 2:**
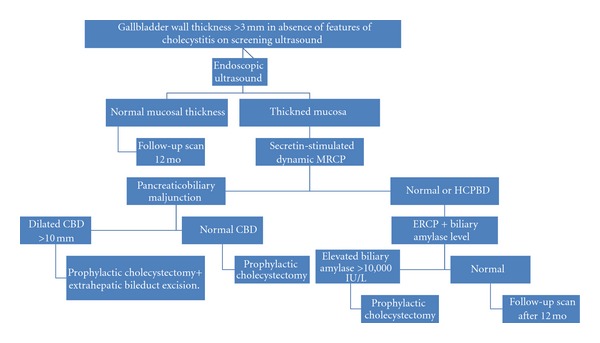
Algorithm for management of thickened gallbladder wall.

The prevalence of gallbladder polyps (Figure 1) varies from 0.3% to 12% in healthy adults who undergo abdominal ultrasonography (US). GB polyps are classified into 2 groups: neoplastic (adenomas, adenocarcinomas) and nonneoplastic (cholesterol polyps, inflammatory polyps, adenomyomatosis). GB polyps larger than 10 mm in diameter are generally indications for cholecystectomy because of the risk of malignancy. The largest PLG series was a review of 172 surgically resected cases, and this showed that the most common type of PLG was the cholesterol polyp (62.8%). They also reported that 7% were inflammatory polyps, 7% were hyperplasia, 5.9% were adenoma, 9.6% were miscellaneous, and 7.7% were malignant polyps in the study population [14].

However, several reports have shown widely varying incidence rates of neoplastic pathologic conditions in 10 to 20 mm (26–88%) [15] and 6 to 10 mm polyps (19–25%) [16]. Thus, an accurate imaging assessment to differentiate neoplastic GB polyps from nonneoplastic ones is required to overcome the limitations of size criteria alone.

 Ultrasound features to be considered in diagnosis of polyps are number (solitary or multiple), size (<6 mm, 6–10 mm, >1 cm), shape (pedunculated or sessile), echogenicity (hypo, iso, and hyper), surface (smooth or nodular), internal echogenicity (homogenous or inhomogeneous), and hyperechoic spots (single 1–5 mm, highly echogenic dot, or partial aggregates of 1–3 mm sized, multiple, highly echogenic spots) [17]. 

Color Doppler ultrasonography has been reported to be useful in the evaluation of malignant lesions. Hirooka et al. [18] reported that in cancerous gallbladder polyps, the color signal pattern was diffuse, becoming linear at the base. Velocity and the resistance index were 39.0 ± 12.4 cm/s and 0.62 ± 0.12, respectively, which was significantly different from control measurements.

Contrast-enhanced ultrasonography has added to accuracy of routine ultrasonography in diagnosis of gallbladder diseases. Hattori et al. [19] reported the usefulness of contrast-enhanced ultrasonography using a galactose-based contrast agent (Levovist, Nippon Schering, Japan) for differential diagnosis of polypoid gallbladder lesions. They examined contrast-enhancement patterns and time-intensity curves. Contrast-enhancement patterns were classified as linear, scattered, diffuse, or branched. When diffuse and branched types were considered indicative of cancer, accuracy was 84.5%, sensitivity 100%, and specificity 76.9%. In gallbladder carcinoma, the time-intensity curve rose sooner than in other diseases as time progressed from no contrast to early phase. In adenocarcinoma, high-intensity values persisted at 120 s. With an intensity of 90 or greater at 120 s taken to indicate cancer, accuracy was 89.7%, sensitivity 89.5%, and specificity 89.7%. The report concluded that ultrasonographic contrast-enhancement patterns show characteristic associations with pathologic findings, serving as a valuable adjunct in diagnosis of gallbladder diseases. 

In a study by Hattori et al. [20], contrast-enhanced ultrasonography using perflubutane (Sonazoid, Daiichi-Sankyo, Tokyo) in evaluating gallbladder carcinoma showed staining throughout the tumor was continuous, consistent with diffuse hypervascularity. Differences between lesion types in flow image size and convection of blood flow were observed. Flowing images, designated as an irregular rolling sign or eruption sign, appeared to be characteristic of gallbladder cancer. On the other hand, in benign gallbladder polyps, staining was scattered with the flow image being uniform and small. Therefore, contrast-enhanced ultrasonography using perflubutane should be useful in the differential diagnosis of gallbladder tumors.

EUS is considered superior to transabdominal US for imaging the biliary system, with higher ultrasound frequencies (5–12 MHz versus 2–5 MHz). EUS can differentiate the double-layered structure of the GB wall and provide higher resolving power for small polypoid lesions.Two EUS scoring systems have been proposed to predict neoplastic GB polyps. Choi et al. [21] suggested a scoring system based on layer structure, echo patterns, the margin of the polyps, and stalk and polyp numbers. At a cut-off score of 6, the sensitivity and specificity for the risk of neoplastic polyps were 4.6% and 84.6%, respectively. Sadamoto et al. [22] proposed another EUS formula: maximum diameter (in millimeters) + internal echo pattern score (heterogenous = 4, homogenous = 0) + hyperechoic spot (5). With this system, the sensitivity and specificity for the risk of neoplastic polyps with scores of >12 were 77.8% and 82.7%, respectively.

In a study by Cho et al. [23], the presence of hypoechoic foci alone allows differentiation between neoplastic and nonneoplastic GB polyps with relatively high sensitivity (90%) and specificity (89%). And polyps from 15 to 20 mm with hypoechoic foci were malignant (sensitivity 85%, specificity 87%, and positive predictive value 85%).

Recently nuclear scans have been studied for evaluating malignant tumor in gallbladder polyps. (18)F-FDG uptake in a GP measured by standardized uptake value and ratio of SUVgp to mean SUV of the liver (GP/L ratio) was high predictor of malignancy [24]. In another study by Nishiyama et al. [25], it was shown that delayed (18)F-FDG PET is more helpful than early (18)F-FDG PET for evaluating malignant lesions because of increased lesion uptake and increased lesion-to-background contrast and also combining it with retention index increased sensitivity to 100% and specificity to 80%. But the limitation of both studies was that in a setting of acute cholecystitis or previous episode of cholecystitis there was high rate of false positivity and a pretest CRP levels assessment can increase the specificity of these tests.

## 5. Pancreaticobiliary Maljunction

Pancreaticobiliary maljunction is defined as an abnormal union of the pancreatic and biliary ducts that is located outside the duodenal wall, where a sphincter system is not present. Thus, two ducts are always communicating, and pancreatic juice freely regurgitates into the biliary tract through this passage. Numerous studies have shown that pancreatobiliary reflux is a major risk factor for biliary carcinogenesis in patients with PBM; the mixture of bile, and pancreatic juice can induce chronic inflammation and genetic alterations and increase cellular proliferation of the biliary tract epithelium, leading to hyperplasia, dysplasia, and ultimately carcinoma of the biliary tract mucosa [26]. 

The risk of gallbladder carcinoma associated with PBM is substantial; it was reported that the occurrence of biliary cancer in 388 PBM patients without biliary dilatation was 37.9%, including 93.2% with gallbladder carcinoma and 6.8% with bile duct cancer, while that in 1239 PBM patients with choledocal cyst was 10.6%, including 33.6% with extrahepatic bile duct cancer, and 64.9% with gallbladder carcinoma [27]. 

Reflux of pancreatic juice into the biliary tract is influenced by the function of Oddies sphincter and the form of the junction of the pancreaticobiliary duct. One of the mechanisms of pancreatobiliary reflux in non-PBM patients could be a long common channel or high confluence of pancreaticobiliary ducts (HCPBD). A common channel length ≥ 6 mm, in which the communication was occluded when the sphincter was contracted. In HCPBD patients, the amylase level in the bile was frequently elevated, and hyperplastic change of the gallbladder epithelium was frequently observed.

Misra et al. [28] reported that a common channel more than 8 mm in length was seen more frequently in patients with gallbladder carcinoma (38%) compared with normal subjects (3%) or patients with gallstones (1%). Kamisawa et al. [29] also reported that the occurrence of gallbladder carcinoma in non-PBM patients with a common channel of more than 6 mm in length was 12%, being significantly higher than that in controls.

 Igarashi [30] reported that gallbladder wall thickening was sometimes observed in PBM cases without biliary dilatation. Epithelial hyperplasia of the gallbladder induced by longstanding continuous stasis of the bile intermingled with refluxed pancreatic juice has been reported to be one of the characteristic pathological changes in PBM patients [31]. The incidence of epithelial hyperplasia of the gallbladder of PBM patients without biliary dilatation was reported to be 72% to 91%. Tanno et al. [32] reported that the Ki-67 labeling index of epithelial hyperplasia of PBM patients was elevated to 6.1%  ± 1.5% and K-ras mutation was detected in 2 (13%) of 15 patients. To detect PBM without biliary dilatation before onset of gallbladder cancer, we should perform MRCP for individuals showing gallbladder wall thickness on US [33]. As gallbladder carcinoma is associated with 35–44% of cases of pancreaticobiliary maljunction without biliary dilatation, prophylactic cholecystectomy is recommended once pancreaticobiliary maljunction is diagnosed [34]. 

Endoscopic ultrasonography (EUS) can detect the confluence of pancreatic duct and bile duct in the proximal portion of the duodenal wall and the so-called common channel PBM often shows a thickness of the inner low echoic layer of the gallbladder, which means histologically mucosal hyperplasia. EUS shows the normal gallbladder wall to be a two-layered structure consisting of an inner hypoechoic layer composed of the mucosa and the muscular layer, and an outer hyperechoic layer composed of the subserosal layer and the serosa. On EUS, the gallbladder wall of patients with PBM appeared as two thickened layers showing epithelial hyperplasia and subserosal fibrosis or three thickened layers containing a middle, more hypoechoic layer showing a hypertrophic muscular layer [35].

Magnetic resonance cholangiopancreatography (MRCP) has become a common non-invasive method for obtaining high quality images of the pancreaticobiliary tree. Reconstruction images on 3D-computed tomography (CT) can also show pancreaticobiliary images. MRCP and 3D-CT can diagnose PBM, based on findings of an anomalous union between the common bile duct and the pancreatic duct, in addition to a long common channel. However, in some cases in which a common channel is not so long and cannot be depicted on MRCP, the MRCP diagnosis of PBM is not possible [36]. 

Diagnostic accuracy can be increased with dynamic MRCP with secretin stimulation or 3-dimensional MRCP. Pancreaticobiliary reflux in PBM patients can be visualized radiologically using secretin-stimulated dynamic MRCP. In normal pancreaticobiliary dynamics, the extrahepatic and intrahepatic bile ducts show no change following secretin injection. On the other hand, in PBM patients, the volume of the extrahepatic bile duct and the gallbladder increases, due to the regurgitation of pancreatic fluid secreted after the injection of secretin into the bile duct [37].

 Endoscopic retrograde cholangiopancreatography (ERCP) is the most effective examination method for close observation of the pattern of the junction site. When the communication between the pancreatic and bile ducts is maintained despite contraction of the sphincter on ERCP, PBM is diagnosed.

 Pancreatography via the minor duodenal papilla can also demonstrate pancreatobiliary reflux in PBM patients. When injected endoscopically via the minor duodenal papilla, the contrast medium is refluxed into the bile duct through a long common channel without outflow into the duodenum [38]. 

Intraductal ultrasonography (IDUS) is performed over the guidewire during the ERCP and is useful for the depiction of the confluence of pancreatic duct and bile duct. IDUS is also useful for the diagnosis of bile duct cancer [39]. However, IDUS has limitations for the diagnosis of bile duct and gallbladder lesions because of shallow US penetration (<2.0 cm) and maneuverability of passage of probe in case of bile duct stricture or a narrow cystic duct.

Pancreatobiliary reflux with extremely high biliary amylase levels and associated gallbladder carcinoma could be identified in patients with and without pancreaticobiliary maljunction, and those patients might be detected by ultrasonography and bile sampling [40]. High bile amylase levels are found in some patients without PBM. Anderson et al. [41] have reported that the bile amylase level obtained through an indwelling T-tube was higher than the serum amylase level in 21 (81%) of 26 patients with biliary tract disease and that bile amylase level fluctuated considerably in the same patient. Noda et al. [40] advocated prophylactic cholecystectomy for patients with high biliary amylase levels without features of PBM due to high risk of gallbladder carcinoma.

## 6. Conclusion 

Gallbladder carcinoma is a lethal tumor with poor prognosis due to delayed presentation and early spread. Early diagnosis and identification of high-risk cases and providing prophylactic cholecystectomy could offer a potential cure for patients. There is a need for mass screening for GBC among population. The use of endoscopic ultrasound and elastography can help early diagnosis of GBC. Pancreaticobiliary reflux is more prevalent than previously thought. Early diagnosis using secretin MRCP and biliary amylase estimation and prophylactic cholecystectomy for high-risk cases can diagnose GBC at early stages and offer a potential cure for patients.
